# Deaf Sign Language Users, Health Inequities, and Public Health: Opportunity for Social Justice

**Published:** 2011-02-15

**Authors:** Steven Barnett, Michael McKee, Scott R. Smith, Thomas A. Pearson

**Affiliations:** Family Medicine Research Program. Dr Barnett is also affiliated with the Rochester Prevention Research Center, National Center for Deaf Health Research; the University of Rochester Department of Family Medicine; and the University of Rochester Department of Community and Preventive Medicine, Rochester, New York; Rochester Prevention Research Center, National Center for Deaf Health Research, University of Rochester Department of Family Medicine, University of Rochester Department of Community and Preventive Medicine, Rochester, New York; Rochester Prevention Research Center, National Center for Deaf Health Research, University of Rochester Department of Community and Preventive Medicine, Rochester, New York; Rochester Prevention Research Center, National Center for Deaf Health Research, University of Rochester Department of Community and Preventive Medicine, Rochester, New York

## Introduction

Inequities in health and health care have increasingly become an area for concern and action for public health professionals, clinicians, policy makers, and communities. Research has documented inequities in the prevalence of chronic diseases of subpopulations defined by education, income, race and ethnicity, and English proficiency. Justice, a cornerstone of medical ethics, calls for corrective actions (1).

We contend that all communities contain a minority group whose health needs are understudied and underserved. This group is the sign language–using deaf population. Most sign language users have been deaf since birth or early childhood (2-7). Sign language is not global nor is it based on a local spoken language. For example, British Sign Language (BSL) and American Sign Language (ASL) are distinct languages with little in common with the English language or each other. In the United States, an estimated 100,000 to 1 million people use ASL as their primary language. We describe 4 issues that underlie health inequities experienced by deaf sign language users and propose 6 public health approaches to address these health and health care inequities to promote health and prevent chronic diseases.

## Four Issues That Underlie Health Inequities Experienced by Deaf Sign Language Users

**Data on the health of deaf persons are lacking.** In *Healthy People 2010*, the absence of health indicator data on groups with disabilities is recurrently cited (8). In the United States, telephone surveys exclude deaf persons, and questionnaires distributed by mail often use written English, a second language for many deaf ASL users (9). Written English surveys may also be inadequate because many people deaf since birth or during childhood have low English literacy (10). The sparse health data that do exist show that adults who have been deaf since birth or early childhood report poorer health than adults in the general US population (11). The lack of the most rudimentary health statistics from deaf populations thwarts efforts to engage deaf communities in setting priorities for health improvement and chronic disease prevention programs.

**Many adults deaf since birth or early childhood have low health literacy**. This low health literacy results from a lifetime of limited access to information that is often considered common knowledge among hearing persons (12). For example, many adults deaf since birth or early childhood do not know their own family medical history, having never overheard their hearing parents discussing this with their doctor (13). Family history is a risk factor for some chronic diseases, including diabetes and heart disease. In the United States, deaf adult sign language users' knowledge of English medical terminology is similar to that of non-English–speaking immigrants to the United States (14). Insufficient knowledge of health-related vocabulary is not limited to deaf adult sign language users with low educational attainment (5). The effect of low health literacy is profound in other minority populations, affecting health care–seeking behaviors, interactions with clinicians, adherence with medical advice, and health outcomes for chronic diseases (15).

**Barriers limit health care with deaf sign language users**. In the United States, adults who have been deaf since birth or early childhood are less likely to have seen a physician than adults in the general population (11). Deaf sign language users are often dissatisfied with physician–patient communication (3,4) and report better access to emergency departments than to primary care (4). Physicians report that deaf patients require more time and effort than hearing patients (16) and that interpreter services are often not available or reimbursed (17). One study showed that deaf ASL users who attended a primary care practice with full-time interpreter services were more likely to report receiving preventive services than a comparison group of deaf ASL users who sought care elsewhere (2). Health care quality indicators do not currently specify deafness or sign language, so the full effect of health care barriers on the health of deaf sign language users and their families is unknown. However, it appears that addressing language barriers improves adherence with some preventive services and may help prevent chronic diseases or improve patients' long-term outcomes through earlier detection.

**Deaf persons may have a biologic basis for some health differences**. In utero or early childhood illnesses (eg, congenital rubella syndrome) that cause deafness may have non-otic sequelae. Heredity likely explains most deafness since birth or early childhood for those born in the United States after the rubella pandemic of the early 1960s. Several genetic conditions involve other organ systems, such as Jervell and Lange-Nielsen syndrome (deafness, a long Q-T interval, and predisposition to arrhythmias and sudden cardiac death) and Usher syndrome (congenital hearing loss and later-onset vision impairment due to retinitis pigmentosa) (Table 1). On the basis of findings from mouse models, emerging research with humans is examining the association of Pendred syndrome (early hearing loss and later-onset euthyroid goiter) with the risk for hypertension and asthma (21). Non-otic effects of other deafness-related genes have not been extensively studied. Although biology explains some health differences experienced by deaf people, their condition-related health outcomes are likely to be worse than those of hearing people with the same condition because of inequities in access to health care, health information, education, and economic resources.

## Six Recommendations for Public Health

**Public health entities must work together with deaf sign language users to address inequities in health information access**. At a minimum, captions or transcripts should be the standard for all publicly available health information that includes audio content. This includes health information videos that target young children so that deaf parents can make informed decisions about the health information content for their hearing children. Captions should be available in English and Spanish, since some Latino ASL users in the United States use Spanish as their second language. Furthermore, efforts should be made to translate health information into ASL and adapt the presentation of that information so that it is culturally appropriate (22). Communities, states, and countries should ensure that their public health emergency communication plans reach deaf people and their families and that emergency workers can access sign language interpreter services. Involving deaf people in the planning and development processes will likely result in better outcomes. Accessible and culturally appropriate health information can help deaf sign language users to make decisions about health and health care behaviors associated with chronic disease risks.

**Include deaf people in surveillance and health research**. Options are required for data collection in the respondent's primary language (ie, ASL for ASL users) as is exploration of ways to include populations excluded by certain survey modalities (eg, telephone surveys). Recruitment strategies need to be adapted because adults who have been deaf since birth or early childhood have little experience with public health research. Recruitment should also be tailored to overcome deaf persons' mistrust of public health professionals that may result from prior negative health care experiences (4). Consent processes should be studied to ensure that researchers and participants have a shared understanding of concepts such as confidentiality, randomization, blinding, and placebo use. Collaborative work to develop accessible survey methods is under way and should continue to include deaf sign language users as partners. Accurate data on health and risks will empower deaf communities to work with public health professionals to establish health priorities, create programs related to those priorities, and evaluate the effectiveness of those programs in preventing chronic diseases.

**Collect new data, and analyze existing data, in ways that allow us to learn about actual deaf populations**. For health data to be useful, surveys should collect deaf-related demographic information (Table 2), such as age at onset of deafness. Adults who have been deaf since before age 3 have different patterns of health care services use (11) and health behaviors (24) than adults who became deaf later in life. This makes sense — a 60-year-old man who has been deaf since birth and a 60-year-old man who has been deaf since age 59 will have different life experiences, including education and employment opportunities and access to health information and health care. It would be surprising if those 2 deaf men had similar health and health care practices. We lose valuable public health information when we conduct analyses that group together all deaf people or all people who are deaf or hard-of-hearing. Reports on analyses of data without deaf-related demographics should acknowledge these limitations. Medical record and billing data currently are limited in their ability to identify people who are deaf (25,26). Establishing standard domains for deaf-related data would allow for meaningful chronic disease surveillance, research, and program evaluation. Data analyses and interpretation of findings should include input from deaf people to enhance relevance and accuracy.

**Encourage deaf sign language users to participate in public health**. Community-based participatory research (CBPR) focused on health, and not specifically hearing, is an example. Deaf sign language users can add content to public health curricula and teach public health students about the deaf community, CBPR, and other topics, including cross-language and cross-cultural issues. To encourage collaboration, public health conferences should facilitate the participation of deaf sign language users. For all conference attendees to benefit from the participation of deaf sign language users, conferences must have interpreter services for presentations, including poster sessions, other formal and informal meetings, and professional networking between conference sessions and in the exhibit halls. Videos shown at conferences and on the Internet must have captions for the audio content.

**Encourage deaf sign language users to pursue careers in public health, health research, and health care**. Deaf sign language users should be encouraged to pursue careers in public health and other health-related fields, as are members of other underserved minority communities. For this effort to be successful, deaf students in health professional programs must have access to the "informal curriculum," aspects of mentoring and professional development that hearing students learn outside of the formal curriculum. These opportunities occur during conversations that take place after meetings and lectures formally end and during impromptu communication with faculty and other students. Public health and other health professional training programs can collaborate with deaf and hearing faculty from other professions to learn from their experiences mentoring deaf learners. Pipeline programs for health careers should expand to reach out to deaf youth. Increasing the number of public health professionals and researchers who are deaf sign language users should enhance collaboration between public health and deaf communities, including dissemination of health information, development of appropriate and accessible programs, and participation in chronic disease research and surveillance. There will be other benefits to increasing the diversity of our public health students, workforce, and faculty. Recruiting from a new demographic may help address the projected shortage of public health professionals (27), as well as facilitate for this underemployed population (28) access to health-related employment (a large and growing segment of the US economy that consists of more than 16 million jobs, representing more than 11% of employed people) (29).

**Advocate for funding to support communication access costs for public health programs and research**. Interpreter services are essential for communication between deaf sign language users and those who are not fluent in sign language. If interpreter services costs are required to come from core program budgets, accessible programs will have fewer resources for their public health initiatives and research. This "penalty" discourages accessibility and creates another disparity. Some mechanisms exist, such as the US Department of Health and Human Services' research supplements to promote diversity in health-related research (PA-08-190). These funding mechanisms are essential to ensure that our chronic disease and other public health–related programs are accessible. To prepare for the increase in demand for sign language interpreter services in these settings, we should start now to advocate for funding to increase the nation's capacity for sign language interpreter services, including advanced training to prepare interpreters for public health–related vocabulary and settings.

## Other Populations

In this article we focus on public health with deaf sign language users. Many deaf and hard-of-hearing people do not know sign language, and they comprise populations that also experience inequities in access to health communication, health care, health research, and health-related careers. Some of our recommendations also apply to other deaf and hard-of-hearing populations, but communication needs are diverse and public health collaboration with these populations will yield additional recommendations to address inequities. We do not attempt to address all of these issues here.

## Conclusion

It has been 20 years since the passage of the Americans with Disabilities Act of 1990, yet deaf sign language users continue to experience inequities accessing health care, health information, health research, and health-related careers, which limits their ability to achieve optimal health for themselves, their families, and their communities. The full effect of these inequities on chronic disease continues to be mostly unmeasured. Bringing about the conditions necessary for people to be healthy is a requirement of social justice (30), as is collecting the data necessary to make that happen (1). Public health has an opportunity to address these inequities and to lead by example by promoting access and collaboration. In this instance, health promotion and chronic disease prevention require social justice, achieved through respectful collaborations to ensure accessible and culturally appropriate communication (31,32), sometimes facilitated with captions and interpreter services.

## Figures and Tables

**Table 1:** Examples of Health Conditions That Have a High Prevalence Among People Who Have Been Deaf Since Birth

**Medical Condition**	Prevalence, General Population	Prevalence, People Deaf Since Birth^c^	Difference
Hereditary long Q-T syndrome	1 in 5,000^a^	3 in 1,000	15 times higher
Retinitis pigmentosa	1 in 4,000^b^	3%-10%	120-400 times higher

a Source: Goldenberg and Moss (18).

b Source: National Library of Medicine (19).

c Source: Barnett et al (20).

**Table 2:** Deaf-Related Demographic Measures Important for Public Health

**Measure^a^**		Examples **a** of How the Measure Is Relevant to Chronic Disease Prevention
Essential measures	Age at onset of deafness	Implications for designing intervention programs, such as those that target families with a deaf child, or school programs with deaf children, or organizations of people with adult-onset deafnessTemporal relationships help identify risk (eg, is being deaf or hard-of-hearing a risk factor for developing diabetes, or is diabetes a risk factor for acquired hearing loss, or are both true?)^b^
	Hearing level (to distinguish deaf, hard-of-hearing, and hearing)	Identifies health and risk behaviors for groups unable to participate in telephone surveillance, such as the current Behavioral Risk Factor Surveillance System (BRFSS)^c^
	Laterality (unilateral or bilateral)	Identifies risks for populations that may have no access to chronic disease prevention interventions that use audio-only format (eg, radio, telephone)
	Preferred communication (including primary language)	Implications for access to health information (in terms of language, modality, and culture)
Potentially important measures	Education setting history (eg, deaf school, integrated programs)	Identifies adult chronic disease risks that may be associated with a childhood education setting, allowing for evidenced-based selection of targeted health promotion interventions for that setting
	Deaf family members	Implications for access to information in the homeImplications regarding genetic risks for chronic diseases
	Usher syndrome (progressive vision loss present in approximately 3%-10% of adults who have been deaf since birth or early childhood)	Identifies chronic disease risks in a population with limited access to audio and visual health promotion and disease prevention messages

a The information presented is not exhaustive.

b Source: Bainbridge et al (23).

c Source: Barnett and Franks (9).

## Appendix. Examining Deaf Population Health Inequities from a Social Justice Perspective (Video)

The videos below are an adaptation of this article featuring dialogue in American Sign Language.
